# Amniotic sac diameter reference interval in early pregnancy between 7 and 10 weeks' gestation

**DOI:** 10.1002/uog.27705

**Published:** 2024-10-30

**Authors:** S. A. Solangon, S. Nijjar, L. V. De Braud, J. Knez, L. Berg, E. Jauniaux, D. Jurkovic

**Affiliations:** ^1^ EGA Institute for Women's Health, Faculty of Population Health Sciences University College Hospital London UK; ^2^ Department for Gynaecology University Medical Centre Maribor Maribor Slovenia

**Keywords:** amniotic sac, early pregnancy, reference interval

## Abstract

**Objective:**

To establish a normal reference interval for amniotic sac diameter (ASD) between 7 + 0 and 9 + 6 weeks' gestation and its relative size in relation to gestational sac diameter (GSD) and the embryo crown–rump length (CRL).

**Methods:**

This was a prospective, cross‐sectional study of consecutive women presenting to the Early Pregnancy Unit, University College Hospital, London, UK, between August 2022 and June 2023. We included live, normally sited, singleton pregnancies with a normal 20‐week anomaly scan. We collected 120 cases per gestational week, from 7 + 0 to 9 + 6 weeks' gestation, totaling 360 cases. We performed an inter‐ and intraobserver variability assessment in the measurement of mean ASD in 30 patients. Regression analyses were used to establish reference intervals for GSD and CRL, ASD and CRL, GSD and ASD, and GSD/ASD ratio and CRL. A fitted regression line was calculated, along with a 90% prediction interval and *R* ^2^ value.

**Results:**

There was good interobserver agreement (mean ± SD difference, 0.007 ± 1.105 mm (95% limits of agreement (LoA), −2.160 to 2.174 mm)) and good intraobserver agreement for Observer A (mean ± SD difference, −0.080 ± 0.741 mm (95% LoA, −1.532 to 1.372 mm)) and Observer B (mean ± SD difference, −0.014 ± 0.919 mm (95% LoA, −1.814 to 1.786 mm)) in the measurement of mean ASD. Regression analyses showed a statistically significant association between each pair of values (*P* < 0.001 for all). There was a significant quadratic association between mean GSD and CRL (*R* ^2^ = 56%), mean GSD and ASD (*R* ^2^ = 60%) and GSD/ASD ratio and CRL (*R* ^2^ = 68%), and a significant cubic association between mean ASD and CRL (*R* ^2^ = 90%). The regression equations were used to quantify the values of ASD and GSD/ASD ratios for a range of CRL values and gestational ages.

**Conclusion:**

Our study has produced comprehensive reference intervals for amniotic sac size in early pregnancy, which could be used in routine clinical practice. © 2024 The Author(s). *Ultrasound in Obstetrics & Gynecology* published by John Wiley & Sons Ltd on behalf of International Society of Ultrasound in Obstetrics and Gynecology.

**CONTRIBUTION**

*What are the novel findings of this work?*

Our study has produced the first comprehensive reference interval for amniotic sac size in early pregnancy, which could be used in routine clinical practice.

*What are the clinical implications of this work?*

Our newly defined reference ranges for amniotic sac diameter in relation to crown–rump length and gestational sac diameter could facilitate earlier and more accurate detection of embryonic abnormalities in the first trimester.

## INTRODUCTION

The amniotic sac develops in the third week after conception from the bilaminar embryonic disk of the implanted blastocyst and subsequently surrounds the developing embryo^1^. At the end of the first trimester, the extraembryonic celom is obliterated gradually by the expanding amniotic sac, which fuses with the placental chorionic plate. Initially, the amniotic fluid is made of embryonic bioproducts that diffuse through the embryonic skin or through the oropharyngeal and cloacal membranes^1^. After 10 weeks' gestation, the fluid electrolyte composition and acid–base balance change rapidly, reflecting fetal kidney development from the mesonephros to the metanephros^2^. During the second and third trimesters, the amniotic fluid is subject to constant turnover, with accumulation of fetal lung fluid and urine and removal by fetal swallowing^2^.

The amniotic cavity becomes visible on transvaginal sonography (TVS) from the 7^th^ week of gestation^3^ but has been less studied in comparison with other early embryonic structures. Robinson and Fleming^4^ were the first to establish normal reference ranges for the first‐trimester crown–rump length (CRL), while a later study also established reference ranges for the gestational sac and yolk sac diameters, and the embryonic heart rate (HR)^5^. However, neither created reference intervals for the amniotic sac. This could be explained partly by difficulties in visualizing the amniotic membrane on TVS.

Recent studies have shown that presence of an amniotic cavity that does not contain a live embryo is a reliable diagnostic sign of early miscarriage^6^. Although an abnormally sized exocelomic cavity and yolk sac could be used to diagnose aneuploid pregnancy and predict miscarriage^7^, ^8^, the potential diagnostic value of assessing the size of the amniotic sac in early pregnancy has not been studied before. Few studies have examined the normal distribution of amniotic sac dimensions, most of which were small and focused on volume measurement^9^, ^10^, ^11^, ^12^, ^13^. The aim of this study was to establish a normal reference interval for amniotic sac diameter (ASD) at 7–10 weeks' gestation and its size in relation to the gestational sac and the embryo CRL. This information could be used in future studies to examine the value of amniotic sac measurement for the diagnosis of early pregnancy abnormalities.

## METHODS

This was a prospective, cross‐sectional study conducted at the Early Pregnancy Unit (EPU), University College Hospital, London, UK, from August 2022 to June 2023. We included consecutive pregnant women at 7 + 0 to 9 + 6 weeks' gestation with a live, eutopic, singleton pregnancy, with a visible amniotic sac on TVS. We included both spontaneous pregnancies and those conceived through assisted reproductive technology (ART). We decided on an upper limit of 10 weeks' gestation for the reference interval, as from then on, transabdominal ultrasound scans can be carried out to diagnose major fetal structural anomalies, and non‐invasive prenatal testing for aneuploidies is readily available. Each woman contributed data from one pregnancy and a single set of measurements.

We excluded pregnancies resulting in miscarriage, termination of pregnancy and pregnancies in which the outcome of the anomaly scan was unknown. We also excluded multiple pregnancies, including those that resulted in a singleton pregnancy due to miscarriage of one or more pregnancies, and pregnancies found to have congenital structural or chromosomal anomaly at the 20‐week anomaly scan.

We were advised by the National Health Service Research Ethics Committee and the Joint Research Office at University College London Hospitals that formal ethical approval was not needed for this study as the data were collected as part of routine care, anonymized and analyzed within the care team. The study was registered with the Research Registry (unique identifying number: researchregistry8168).

Clinical information was obtained as part of routine clinical practice, including maternal age, gravidity, parity, mode of conception, menstrual cycle length and regularity, and indication for presentation^14^. All patients underwent TVS performed using high‐end ultrasound equipment (Voluson E8, GE Medical Systems, Zipf, Austria). Ultrasound examinations were performed by clinical fellows, who were all Level‐II ultrasound operators^14^. They were supervised by five consultant gynecologists with a special interest in early pregnancy care, who were all Level‐III ultrasound operators. Examinations were carried out in a standardized way to ensure that all relevant measurements were obtained and recorded.

A live normally sited pregnancy was defined by the presence of a gestational sac within the uterine cavity, which contained an embryo with visible cardiac activity. In all cases, the following structures were routinely examined: gestational sac (a spherical structure within the uterine cavity, surrounded by the echogenic trophoblast), yolk sac (a small spherical structure within the gestational sac) and amniotic sac (a spherical thin‐walled structure within the gestational sac and distinct from the yolk sac, in which the embryonic pole was situated).

**Figure 1:**
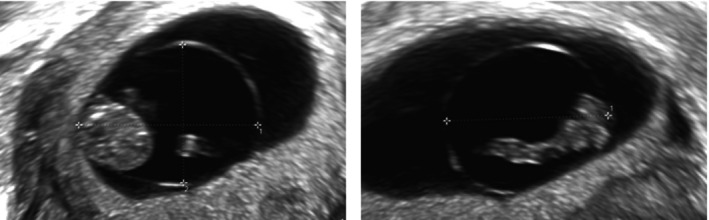
Ultrasound images of the amniotic sac at 8 weeks' gestation, showing three perpendicular measurements which were obtained in the longitudinal and transverse planes.

Measurements were obtained using only two‐dimensional (2D) images, while three‐dimensional (3D) volumes were used to assess inter‐ and intraobserver variability. Mean gestational sac diameter (GSD) was calculated as the average of three perpendicular diameters with the calipers placed at the inner edges of the chorionic cavity^15^. Mean ASD was calculated as the average of three perpendicular diameters with the calipers placed at the inner edges of the amniotic sac wall (Figure 1). Embryonic HR was calculated in bpm using M‐mode. CRL was measured in the sagittal plane of the embryo^16^. Gestational age (GA) was derived from the CRL measurement, using the formula described by Robinson and Fleming^4^.

Clinical information and ultrasound images were stored on dedicated hospital clinical databases (GE HealthCare ViewPoint, version 5.6.25.283) as per our routine practice, and study data were collected only by members of the clinical care team. Data were anonymized and stored securely according to General Data Protection Regulations.

The primary objectives were to determine reference intervals for mean ASD and GSD/ASD ratio, according to CRL. Further analyses, added as supplementary information, included reference intervals for mean ASD, GSD and embryonic HR according to GA, as measured from the last menstrual period (LMP) or *in‐vitro* fertilization (IVF) conception dates, as these may be useful in routine clinical practice.

### Assessment of inter‐ and intraobserver variability

Inter‐ and intraobserver variability was assessed in a subgroup of 30 patients. Real‐time ultrasound examinations were performed, and 3D volumes were obtained. The depth of the acquired volume was adjusted to cover the entire uterus, containing the gestational sac. During volume acquisition, the probe was held steady, and patients were asked to hold their breath. The rendered volumes were saved on the ultrasound machine's hard drive and were examined independently by two operators (Observer A and Observer B). Three measurements of the amniotic sac were taken in longitudinal and transverse reformatted sections to assess interobserver variability. All measurements were recorded by a third investigator who did not participate in the reproducibility analysis. Operators were therefore blinded to their own and each other's measurements. To assess intraobserver variability, operators then re‐examined the stored 3D volumes and repeated the measurements after a minimum of 1 hour after the first examination. The order of examinations was determined randomly by the third investigator to minimize the risk of bias.

Inter‐ and intraobserver agreements were assessed using the Bland–Altman limits of agreement method due to the continuous nature of the measurements. This method predicts the differences likely to occur between pairs of measurements. The mean difference was obtained by calculating the difference between repeat measurements for each patient. The 95% limits of agreement (within which 95% of all differences between values should occur) were then calculated according to the following equation: mean difference ± 1.96 × (SD of differences).

### Sample size

Recommendations suggest that measurements are obtained from a minimum of 120^17^ to 200 individuals, with at least 20 patients per gestational week^18^, ^19^. Our aim was to recruit 120 consecutive cases in each of the 7^th^, 8^th^ and 9^th^ gestational weeks, amounting to 360 individual patients in total.

### Statistical analysis

Statistical analysis of the collected data was performed using Stata (StataCorp LLC, College Station, TX, USA). The baseline variables for normality of distribution were tested using the Shapiro–Wilk test. Normally distributed values are expressed as mean ± SD with 95% limits of agreement. Non‐normally distributed values are expressed as median and interquartile range (IQR). Descriptive data for categorical variables are presented as *n* (%). Reference intervals were generated using the method described by Royston and Wright^20^. All data were assessed using linear regression analysis. Where the assumptions of linear regression were not met (e.g. non‐normally distributed residuals, a fitted value/residual relationship), either one or both variables were analyzed on the log scale. Where required, a small constant was added to all values for a particular variable to facilitate the log transformation. The shape of the relationship between variables was examined. Cubic and/or quadratic terms for the predictor variable were included in the analysis when they improved the fit of the regression model. A regression line was calculated and fitted, along with a 90% prediction interval. The strength of the relationship between variables was also quantified by calculating the *R* ^2^ value.

## RESULTS

**Figure 2:**
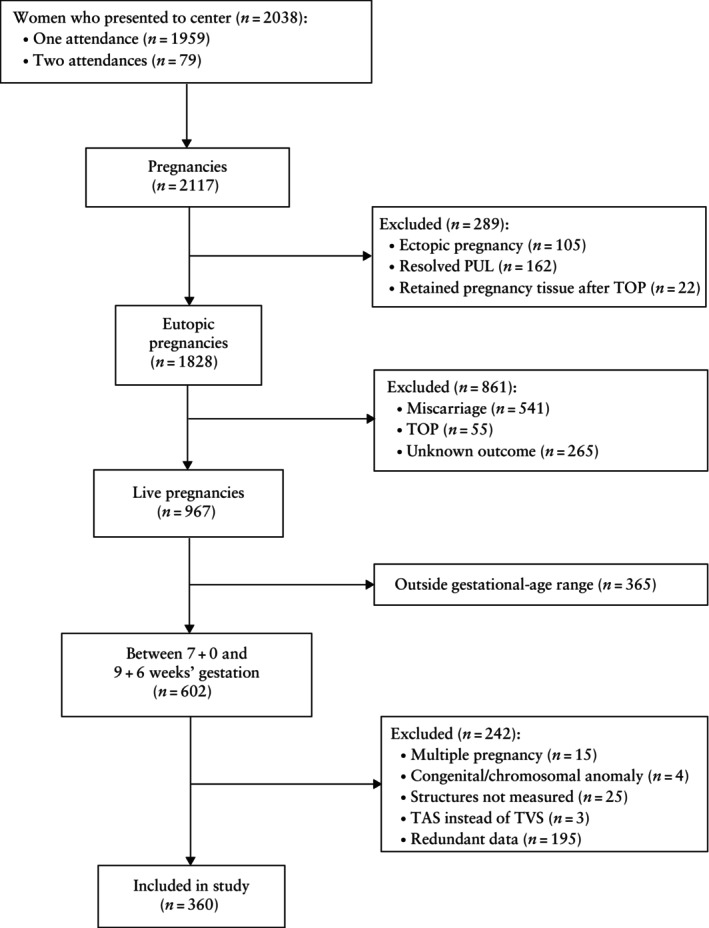
Flowchart summarizing inclusion in the study of singleton pregnancies at 7 + 0 to 9 + 6 weeks' gestation. PUL, pregnancy of unknown location; TAS, transabdominal sonography; TOP, termination of pregnancy; TVS, transvaginal sonography.

During the study period, 2038 women presented to the EPU with a total of 2117 pregnancies. A total of 360 live pregnancies between 7 + 0 and 9 + 6 weeks' gestation were included in the study. The flowchart shown in Figure 2 summarizes the inclusion of women in the study. There was an equal distribution of 120 cases per included gestational week. Maternal characteristics are shown in Table 1. The CRL was measured in all cases. Mean GSD and ASD were obtained in all cases, but embryonic HR measurement was missing in four cases. In 331 of 360 (92%) cases with a known final pregnancy outcome, no aneuploidies were diagnosed at birth.

**Table 1:** Demographic characteristics of 360 women with live singleton pregnancy between 7 + 0 and 9 + 6 weeks' gestation

Characteristic	Value
Maternal age (years)	33 (30–36)
Gestational age (days)	59 (54–64)
Gravidity	
1	92 (25.6)
2	97 (26.9)
3	86 (23.9)
≥ 4	85 (23.6)
Parity	
0	192 (53.3)
1	104 (28.9)
≥ 2	64 (17.8)
Mode of conception	
Spontaneous	321 (89.2)
ART	39 (10.8)
Indication for presentation	
Abdominal pain only	87 (24.2)
Vaginal bleeding only	48 (13.3)
Both pain and bleeding	137 (38.1)
Reassurance	66 (18.3)
Other	22 (6.1)

Data are given as median (interquartile range) or *n* (%).

ART, assisted reproductive technology.

### Inter‐ and intraobserver variability in amniotic sac measurements

**Figure 3:**
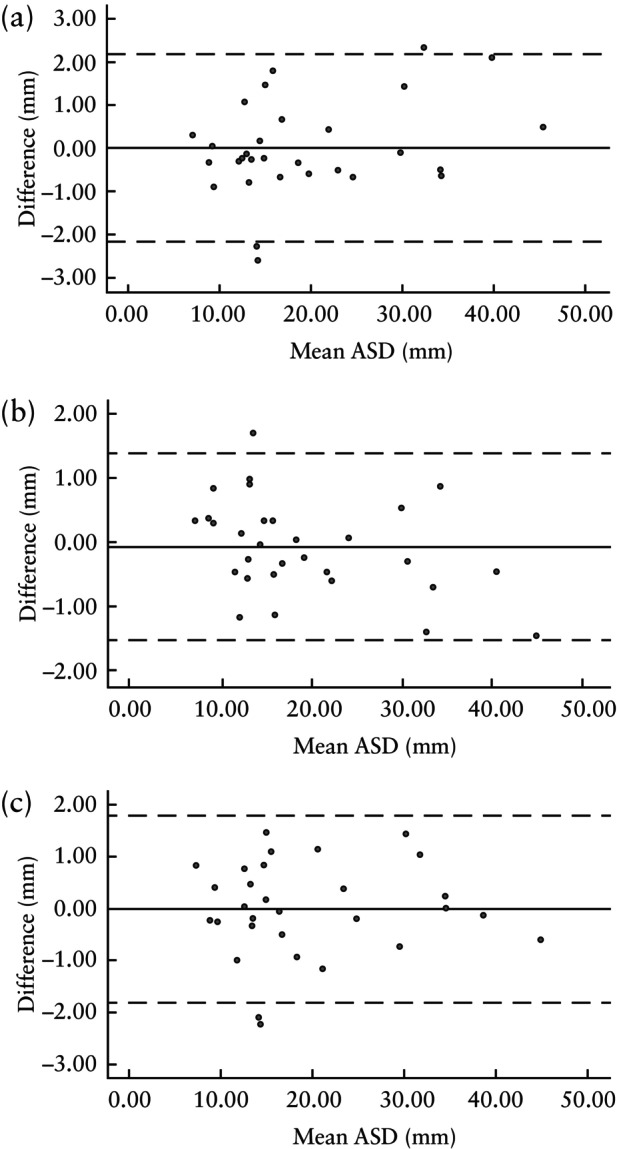
Bland–Altman plots showing mean difference (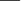) with 95% limits of agreement (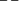) for interobserver variability (a) and intraobserver variability for Observer A (b) and Observer B (c) for measurement of mean amniotic sac diameter (ASD).

Inter‐ and intraobserver agreement in the measurement of mean ASD in a subset of 30 patients is summarized in Table 2. There was good interobserver agreement, reflected by a small mean difference between repeat measurements obtained by the two observers (mean ± SD difference, 0.007 ± 1.105 mm (95% limits of agreement, −2.160 to 2.174 mm)). The differences between the measurements obtained by Observer A (mean ± SD difference, −0.080 ± 0.741 mm (95% limits of agreement, −1.532 to 1.372 mm)) and Observer B (mean ± SD difference, 0.014 ± 0.919 mm (95% limits of agreement, −1.814 to 1.786 mm)) at two different timepoints (intraobserver agreement) also showed good agreement. Bland–Altman plots with 95% limits of agreement for inter‐ and intraobserver variability are displayed in Figure 3.

**Table 2:** Interobserver and intraobserver variability for measurement of mean amniotic sac diameter in 30 cases

Agreement	Difference between measurements (mm)
Interobserver	0.007 ± 1.105 (−2.160 to 2.174)
Intraobserver	
Observer A	−0.080 ± 0.741 (−1.532 to 1.372)
Observer B	−0.014 ± 0.919 (−1.814 to 1.786)

Data are given as mean ± SD (95% limits of agreement).

### Regression equations

A summary of the relationship between each pair of variables is given in Table 3. For each relationship, all regression coefficients are reported, along with corresponding 95% CI, *P*‐values and *R* ^2^ values. The results show a highly statistically significant association between each pair of variables (*P* < 0.001 for all). Although all relationships were highly statistically significant, some relationships were stronger than others. The strongest relationship was between mean ASD and CRL (*R* ^2^ = 90%).

**Table 3:** Summary of regression equations

Outcome	Predictor	Term	Coefficient (95% CI)	*P*	*R* ^2^
Mean GSD	CRL	Constant	10.3 (5.2 to 15.3)	< 0.001	56%
		Linear	1.51 (0.95 to 2.08)		
		Quadratic	−0.017 (−0.032 to −0.002)		
Mean ASD	CRL	Constant	10.5 (2.0 to 19.0)	< 0.001	90%
		Linear	−1.28 (−2.74 to 0.17)		
		Quadratic	0.13 (0.06 to 0.21)		
		Cubic	−0.002 (−0.004 to −0.001)		
Mean GSD	Mean ASD	Constant	15.1 (12.1 to 18.1)	< 0.001	60%
		Linear	1.15 (0.82 to 1.48)		
		Quadratic	−0.011 (−0.019 to −0.003)		
GSD/ASD ratio*	CRL	Constant	1.73 (1.55 to 1.91)	< 0.001	68%
		Linear	−0.083 (−0.104 to −0.063)		
		Quadratic	0.0011 (0.0006 to 0.0017)		

* Variable analyzed on log scale (base e).

ASD, amniotic sac diameter; CRL, crown–rump length; GSD, gestational sac diameter.

**Figure 4:**
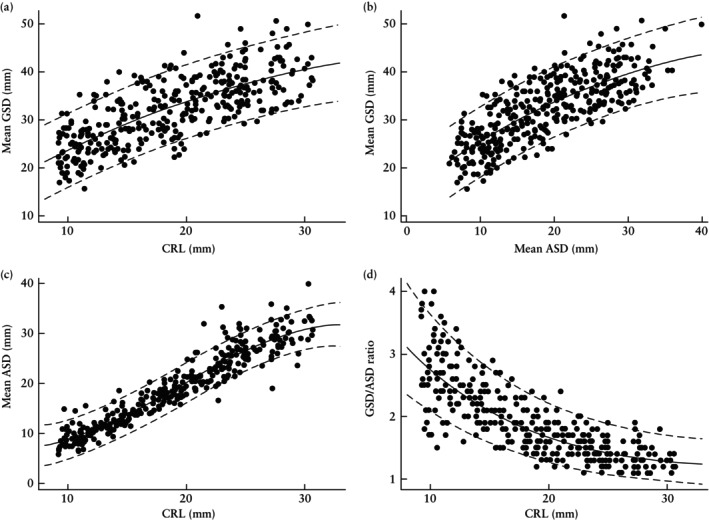
Fitted relationships between mean gestational sac diameter (GSD) and crown–rump length (CRL) (a), mean GSD and mean amniotic sac diameter (ASD) (b), mean ASD and CRL (c) and GSD/ASD ratio and CRL (d). Regression line (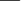) and 90% prediction interval (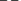) are shown.

There was a significant quadratic association between mean GSD and CRL (*R* ^2^ = 56%), and mean GSD and mean ASD (*R* ^2^ = 60%) (Figure 4a,b). There was a significant cubic association between mean ASD and CRL (*R* ^2^ = 90%) (Figure 4c) and a significant quadratic association between GSD/ASD ratio and CRL (*R* ^2^ = 68%) (Figure 4d). Regression lines and 90% prediction intervals for mean GSD, mean ASD and GSD/ASD ratio according to GA, as calculated using LMP or IVF dates, are shown in Table S1 and Figures [Link], [Link]. Regression equations were also used to quantify the values of ASD and GSD/ASD ratios for a range of CRL values and GA, shown in Tables 4 and 5.

**Table 4:** Estimated amniotic sac diameter (ASD) and gestational sac diameter (GSD)/ASD ratio according to crown–rump length (CRL)

	ASD (mm)			GSD/ASD ratio		
CRL	5^th^ per	50^th^ per	95^th^ per	5^th^ per	50^th^ per	95^th^ per
8 mm	3.6	7.7	11.7	2.35	3.11	4.13
9 mm	4.2	8.2	12.1	2.21	2.92	3.87
10 mm	4.9	8.8	12.7	2.08	2.75	3.63
11 mm	5.7	9.6	13.5	1.96	2.59	3.42
12 mm	6.6	10.5	14.4	1.85	2.44	3.23
13 mm	7.6	11.5	15.4	1.75	2.31	3.05
14 mm	8.7	12.6	16.5	1.66	2.19	2.90
15 mm	9.8	13.7	17.7	1.58	2.09	2.75
16 mm	11.1	15.0	18.9	1.51	1.99	2.62
17 mm	12.3	16.2	20.1	1.44	1.90	2.51
18 mm	13.6	17.5	21.5	1.38	1.82	2.40
19 mm	15.0	18.9	22.8	1.32	1.74	2.30
20 mm	16.3	20.2	24.1	1.27	1.68	2.21
21 mm	17.6	21.5	25.4	1.22	1.62	2.13
22 mm	18.9	22.8	26.7	1.18	1.56	2.06
23 mm	20.2	24.1	28.0	1.14	1.51	1.99
24 mm	21.4	25.3	29.2	1.11	1.47	1.93
25 mm	22.6	26.5	30.4	1.08	1.42	1.88
26 mm	23.6	27.6	31.5	1.05	1.39	1.83
27 mm	24.6	28.5	32.5	1.03	1.36	1.79
28 mm	25.5	29.4	33.3	1.00	1.33	1.76
29 mm	27.4	31.6	35.8	0.94	1.25	1.66
30 mm	27.2	31.3	35.4	0.97	1.28	1.70
31 mm	26.8	30.8	34.8	0.93	1.24	1.65
32 mm	27.4	31.8	36.2	0.98	1.30	1.72
33 mm	26.2	30.2	34.1	0.95	1.26	1.68

Per, percentile.

**Table 5:** Estimated amniotic sac diameter (ASD) and gestational sac diameter (GSD)/ASD ratio according to gestational age (GA)

	ASD (mm)			GSD/ASD ratio		
GA	5^th^ per	50^th^ per	95^th^ per	5^th^ per	50^th^ per	95^th^ per
49 days	3.8	7.8	11.9	2.15	2.85	3.79
50 days	4.7	8.7	12.7	2.04	2.71	3.59
51 days	5.6	9.6	13.6	1.94	2.57	3.41
52 days	6.6	10.6	14.6	1.85	2.45	3.24
53 days	7.6	11.6	15.6	1.76	2.33	3.09
54 days	8.6	12.6	16.6	1.68	2.22	2.94
55 days	9.7	13.7	17.7	1.60	2.12	2.81
56 days	10.8	14.8	18.8	1.53	2.03	2.68
57 days	11.9	15.9	19.9	1.46	1.94	2.57
58 days	13.1	17.1	21.1	1.40	1.86	2.46
59 days	14.3	18.3	22.3	1.34	1.78	2.36
60 days	15.6	19.6	23.6	1.29	1.71	2.27
61 days	16.8	20.9	24.9	1.24	1.64	2.18
62 days	18.2	22.2	26.2	1.19	1.58	2.10
63 days	19.5	23.5	27.5	1.15	1.52	2.02
64 days	20.9	24.9	28.9	1.11	1.47	1.95
65 days	22.4	26.4	30.4	1.07	1.42	1.88
66 days	23.8	27.8	31.9	1.04	1.37	1.82
67 days	25.3	29.3	33.4	1.00	1.33	1.77
68 days	26.8	30.9	34.9	0.97	1.29	1.71
69 days	28.4	32.5	36.5	0.94	1.25	1.67

Per, percentile.

## DISCUSSION

### Main findings

We have collected sufficient data to establish reference intervals for ASD in live, normally sited pregnancies between 7 and 10 weeks' gestation. In all pregnancies, the amniotic sac was visualized clearly and all measurements were successfully and accurately recorded with good inter‐ and intraobserver variability. We have provided charts showing mean ASD and GSD/ASD ratio according to CRL and GA, which are easy to use and can be incorporated into routine clinic practice.

### Strengths and limitations

Our study followed stringent methodological quality criteria^21^ to develop a high‐quality reference interval for ASD measurements in early pregnancy. We analyzed the association of parameters with CRL, as previous reports recommend the use of CRL measurements for establishing GA‐related reference intervals rather than LMP, which is subject to variability due to the irregularity of menstrual cycles and uncertainty of menstrual dates^5^. We only included pregnancies resulting in a live birth and without congenital structural or chromosomal abnormalities, detecting up to and including the 20‐week anomaly scan. One case was a consistent outlier with a longer mean GSD, but nuchal scan findings were normal, and the patient delivered a healthy neonate at term. In view of this, the case was not excluded from the reference interval, as advised by Altman and Chitty^22^.

A potential limitation of our study is the inclusion of symptomatic patients; however, we only included pregnancies resulting in a live birth. We also had incomplete follow‐ups of patients after 20 weeks' gestation. However, the potential benefits of establishing a reference range for ASD between 7 to 10 weeks' gestation include a better assessment of miscarriage risk in live pregnancies and improved early detection of embryonic anomalies, including aneuploidies, prior to a routine first‐trimester anomaly scan performed from 10 weeks' gestation^23^.

Our study included both ART and spontaneously conceived pregnancies. Previous studies have reported both shorter and longer CRL in ART pregnancies compared with that in spontaneously conceived pregnancies^24^, ^25^, ^26^, ^27^. A systematic review has highlighted both underestimation and overestimation of CRL measurements between ART and spontaneous conceptions, leading to conflicting results in early pregnancies of < 51 days' gestation^28^. As we only included pregnancies at 7–10 weeks' gestation, these reported variations should not affect the accuracy of CRL measurements in our study.

We found a highly significant positive association between mean ASD and CRL with an *R* ^2^ value of 90%. At 7–9 weeks' gestation, this is likely due to the accumulation of early embryonic bioproducts^1^, whereas from 9 weeks' gestation, the rapid expansion of the amniotic cavity is associated with the development of nephrons and definitive kidneys^2^. This explains the decrease in GSD/ASD ratio as CRL increases (*R* ^2^ = 68%). An increase in amniotic fluid volume (AFV) as CRL increases indicates normal embryonic development during organogenesis, and a discrepancy in this could be a sign of an underlying embryonic abnormality. Oligohydramnios with chorioamniotic separation in the second trimester has been reported to be associated with triploidy, fetal congenital anomalies and major structural malformations, such as renal agenesis, pulmonary airway malformation and cardiac anomalies^29^, ^30^, ^31^. There are a few anomalies that can be detected in the first trimester, including body stalk anomaly (BSA). BSA is typically associated with amniotic membrane abnormality and routine amniotic sac examination could facilitate its early detection^32^. However, literature on congenital anomalies and associated oligohydramnios in the first trimester is sparse^33^, ^34^, highlighting future research potential and the clinical relevance of establishing reference intervals for ASD.

Head‐to‐trunk measurement ratios in the first trimester have been used to predict aneuploidies^35^. In our center, we have seen pregnancies with selective reduction of the exocelomic cavity and low GSD/ASD ratio, which were associated with chromosomal abnormality. The relationship between GSD/ASD ratio and CRL in our study showed a narrow 90% prediction interval range, which could be used to detect disproportion in gestational sac and amniotic sac sizes. This could facilitate earlier detection of abnormal pregnancies and better counseling of patients.

Various studies have reported on the roles of the gestational sac, yolk sac, CRL and embryonic cardiac activity to predict adverse outcomes in early pregnancy^36^, ^37^, ^38^, ^39^, ^40^. Other studies have reviewed a combination of ultrasound and demographic variables to create risk prediction models for pregnancy outcomes^8^, ^41^, ^42^, ^43^. So far, none of the proposed models have reached the level of accuracy for the prediction of miscarriage required for use in routine clinical practice. There has been no research regarding the discrepancies in the size of amniotic fluid compartments in early pregnancy to predict adverse outcomes.

Advances in technology and the use of TVS for greater resolution power enable visualization and accurate measurement of the amniotic sac, as demonstrated in our study. The few studies conducted on first‐trimester amniotic sac biometry have focused mostly on assessing AFV. Weissman *et al*.^9^ measured AFV in 95 pregnant women with 2D ultrasound images using the ellipsoid model and found a good positive correlation between GA and AFV (*R* ^2^ = 78%). Similarly, smaller studies using 3D sonographic volumetry found a significant association between amniotic sac volume (ASV) and GA, and ASV and CRL^10^, ^11^, ^12^. However, 3D ultrasound imaging may be less accessible and more complex than 2D imaging, and it appears to add little diagnostic and prognostic value compared with 2D imaging^44^, ^45^. Others argue that sections reconstructed from ultrasound volumes may be less accurate than 2D measurements^13^. The ASV interval also widens as pregnancy advances, making it less valuable for detecting discrepancies. One study of women between 5 and 12 weeks' gestation, of which 193 had a visible amniotic sac on TVS, found a significant correlation between ASD and GA (*R* ^2^ = 74%), and ASD and CRL (*R* ^2^ = 90%), similar to the results in our study (GA, *R* ^2^ = 79%; CRL, *R* ^2^ = 90%), although it was not specified how ASD was measured or whether this was a single measurement or a mean of multiple measurements^13^.

### Implications for clinical practice and future research

ASD is not routinely measured in early pregnancy, possibly due to a lack of evidence demonstrating its clinical relevance in the prediction or diagnosis of early pregnancy complications. However, previous studies have shown that the ultrasound finding of an amniotic sac in the absence of a live embryo is an accurate predictor of miscarriage^46^, ^47^, with one large prospective study reporting this finding to have 100% specificity and 100% positive predictive value for pregnancy failure^6^. Close examination of the amniotic sac in early pregnancy can also help detect anomalies incompatible with life, such as BSA. Further studies are needed to determine whether amniotic sac size discrepancies or new algorithms can predict adverse pregnancy outcomes reliably. We have demonstrated the ease and accuracy of measuring ASD, and this measurement should be incorporated into routine early‐pregnancy ultrasound assessments.

To conclude, our study has produced comprehensive reference intervals for ASD in early pregnancy, which could be used in routine clinical practice. Further research is needed to investigate the potential diagnostic value of amniotic and gestational cavity size discrepancies for the prediction of miscarriage and early detection of embryonic and fetal anomalies.

## Supporting information


**Figure S1** Fitted relationship between mean gestational sac diameter (GSD) and gestational age (GA). Regression line and 90% prediction interval are shown.

**Figure S2** Fitted relationship between mean amniotic sac diameter (ASD) and gestational age (GA). Regression line and 90% prediction interval are shown.

**Figure S3** Fitted relationship between gestational sac diameter (GSD) to amniotic sac diameter (ASD) ratio and gestational age (GA). Regression line and 90% prediction interval are shown.

**Table S1** Summary of regression equations according to gestational age (GA)

## Data Availability

The data underlying this article will be shared on reasonable request to the corresponding author.
