# Supplementary figures and images for: Amniotic sac diameter reference interval in early pregnancy between 7 and 10 weeks' gestation
Source: Ultrasound Obstet Gynecol. 2024 Oct 30;64(6):799–807. doi: 10.1002/uog.27705 (PMC11610665; doi:10.1002/uog.27705)

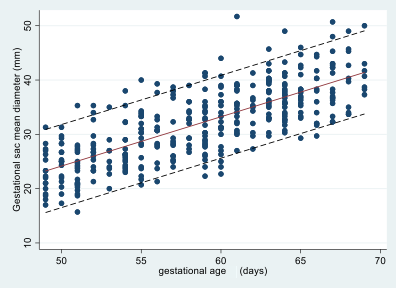

Supplement: Supplementary file 1 — Figure S1 Fitted relationship between mean gestational sac diameter (GSD) and gestational age (GA). Regression line and 90% prediction interval are shown. [file UOG-64-799-s003.png]

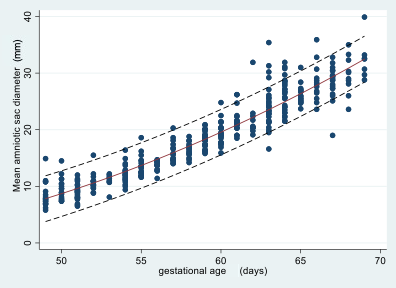

Supplement: Supplementary file 2 — Figure S2 Fitted relationship between mean amniotic sac diameter (ASD) and gestational age (GA). Regression line and 90% prediction interval are shown. [file UOG-64-799-s004.png]

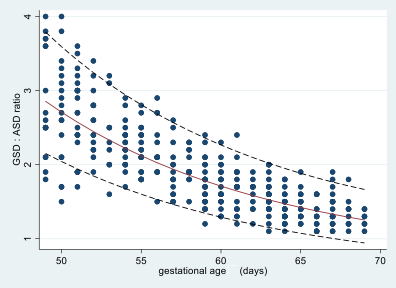

Supplement: Supplementary file 3 — Figure S3 Fitted relationship between gestational sac diameter (GSD) to amniotic sac diameter (ASD) ratio and gestational age (GA). Regression line and 90% prediction interval are shown. [file UOG-64-799-s002.png]
