# Supplementary material for: Amniotic sac diameter reference interval in early pregnancy between 7 and 10 weeks' gestation
Source: Ultrasound Obstet Gynecol. 2024 Oct 30;64(6):799–807. doi: 10.1002/uog.27705 (PMC11610665; doi:10.1002/uog.27705)
Supplement: Supplementary file 4 — Table S1 Summary of regression equations according to gestational age (GA) [file UOG-64-799-s001.docx]

**SUPPLEMENTARY TABLE**

***Table S1*** *Summary of regression equations according to GA in days*

| Outcome | Predictor | Term | Coefficient  (95% CI) | P-value | R^2^ |
| --- | --- | --- | --- | --- | --- |
| GSD | GA | Constant | -21.2 (-26.2, -16.3) | <0.001 | 56% |
|  |  | Linear | 0.91 (0.82, 0.99) |  |  |
| Mean ASD | GA | Constant | 9.27 (-19.0, 37.6) | <0.001 | 89% |
|  |  | Linear | -0.93 (-1.90, 0.05) |  |  |
|  |  | Quadratic | 0.018 (0.010, 0.27) |  |  |
| GSD:ASD ratio ^(*)^ | GA | Constant | 5.1 (3.1, 7.1) | <0.001 | 67% |
|  |  | Linear | -0.11 (-0.18, -0.04) |  |  |
|  |  | Quadratic | 0.0006 (0.0000, 0.0012) |  |  |

(*) Variable analysed on the log scale (base e). ASD, amniotic sac diameter; GA, gestational age; GSD, gestational sac diameter.
